# Effectiveness of Individual Real-Time Video Counseling on Smoking, Nutrition, Alcohol, Physical Activity, and Obesity Health Risks: Systematic Review

**DOI:** 10.2196/18621

**Published:** 2020-09-11

**Authors:** Judith Byaruhanga, Prince Atorkey, Matthew McLaughlin, Alison Brown, Emma Byrnes, Christine Paul, John Wiggers, Flora Tzelepis

**Affiliations:** 1 School of Medicine and Public Health University of Newcastle Callaghan Australia; 2 Hunter New England Population Health Wallsend Australia; 3 Hunter Medical Research Institute New Lambton Heights Australia; 4 Priority Research Centre for Health Behaviour Faculty of Health & Medicine University of Newcastle Callaghan Australia

**Keywords:** telehealth, videoconferencing, smoking cessation, diet, alcohol drinking, physical activity, obesity, mobile phone

## Abstract

**Background:**

Real-time video communication technology allows virtual face-to-face interactions between the provider and the user, and can be used to modify risk factors for smoking, nutrition, alcohol consumption, physical activity, and obesity. No systematic reviews have examined the effectiveness of individual real-time video counseling for addressing each of the risk factors for smoking, nutrition, alcohol consumption, physical activity, and obesity.

**Objective:**

This systematic review aims to examine the effectiveness of individually delivered real-time video counseling on risk factors for smoking, nutrition, alcohol consumption, physical activity, and obesity.

**Methods:**

The MEDLINE (Medical Literature Analysis and Retrieval System Online), EMBASE (Excerpta Medica Database), PsycINFO, Cochrane Register of Controlled Trials, and Scopus databases were searched for eligible studies published up to November 21, 2019. Eligible studies were randomized or cluster randomized trials that tested the effectiveness of individual real-time video communication interventions on smoking, nutrition, alcohol, physical activity, and obesity in any population or setting; the comparator was a no-intervention control group or any other mode of support (eg, telephone); and an English-language publication.

**Results:**

A total of 13 studies were eligible. Four studies targeted smoking, 3 alcohol, 3 physical activity, and 3 obesity. In 2 of the physical activity studies, real-time video counseling was found to significantly increase physical activity when compared with usual care at week 9 and after 5 years. Two obesity studies found a significant change in BMI between a video counseling and a documents group, with significantly greater weight loss in the video counseling group than the in-person as well as the control groups. One study found that those in the video counseling group were significantly more likely than those in the telephone counseling group to achieve smoking cessation. The remaining studies found no significant differences between video counseling and telephone counseling or face-to-face counseling for smoking cessation, video counseling and face-to-face treatment on alcohol consumption, video counseling and no counseling for physical activity, and video counseling and face-to-face treatment on BMI. The global methodological quality rating was moderate in 1 physical activity study, whereas 12 studies had a weak global rating.

**Conclusions:**

Video counseling is potentially more effective than a control group or other modes of support in addressing physical inactivity and obesity and is not less effective in modifying smoking and alcohol consumption. Further research is required to determine the relative benefits of video counseling in terms of other policy and practice decision-making factors such as costs and feasibility.

## Introduction

### Background

Tobacco use, poor nutrition, risky alcohol consumption, physical inactivity, and obesity are the leading modifiable health risks that can cause noncommunicable diseases, including cardiovascular disease, chronic respiratory disease, cancer, stroke, and diabetes [1]. Globally, it is estimated that there are 1.1 billion tobacco smokers, and tobacco use is responsible for the death of 8 million people each year [2]. Harmful alcohol consumption is responsible for 3 million deaths and causes >200 chronic and acute diseases [3]. Globally, in 2016, an estimated 0.9 million injury deaths and 52.4 million injury disability-adjusted life years (DALYs) were attributable to alcohol [3]. Similarly, poor diet accounts for 11 million adult deaths [4], of which 3 million annual deaths are attributed to excess salt or sodium intake [5], 2 million deaths per year are attributable to diets low in fruits and vegetables, and 3 million deaths are attributable to low intake of whole grains [4,6]. Insufficient physical activity causes 5.3 million premature deaths annually [7].

Real-time video communication, also known as videoconferencing, telehealth, or telecare [8], is a scalable and accessible intervention delivered over the internet via a video camera connected to a computer, smartphone, or tablet [9]. Real-time video communication is available to the 3.9 billion people who have access to the internet worldwide [10] and have a device with a video camera. Video communication software such as Skype can be downloaded for free by internet users and is widely used for personal or professional communication every day [11]. Real-time video technology allows a real-time, virtual face-to-face interaction between the provider and the user [12] at any time of the day in any location with internet access [13]. Another advantage of real-time video counseling is that it provides a mode for delivering individual counseling that allows counselors to respond to the client’s verbal and nonverbal cues, unlike telephone support, which is an audio-only intervention, or written materials. Real-time video counseling is supported by the media richness theory, which conceptualizes that real-time video counseling ranks highly as a rich mode of communication as it provides virtual face-to-face support and advisers are able to respond to nonverbal cues, which is not possible in all forms of behavioral support [14]. There is an opportunity for instant clarification of doubts or identifying reluctance or enthusiasm in both the voice and body language, consequently minimizing chances of being misunderstood. Real-time video counseling also eliminates travel time and associated costs of face-to-face interventions [15], improves discretion and comfort as the video call can be taken in a preferred private place to avoid the potential stigma associated with clinic visits [15,16], and has widespread reach. Real-time video counseling has the potential to be used on a large scale to target health risks attributable to smoking, nutrition, alcohol consumption, physical activity, and obesity [9,10].

Individual counseling is used by service providers to deliver support for smoking cessation [17], nutrition [18], physical activity [19], obesity [19], and alcohol consumption [20]. Individual counseling is primarily delivered in person or via the telephone by service providers [21-24], and these modes have been found to be effective in improving health risks attributable to smoking, nutrition, alcohol consumption, physical activity, and obesity [17-19,25-27]. The advantages of individual counseling include convenient scheduling for both the client and service provider, focused treatment with personalized feedback, and a high level of confidentiality as clients can disclose themselves in private compared with group counseling [28].

The capability, opportunity, motivation, and behavior (COM-B) model by Michie et al [29] provides a theoretical framework to examine the effectiveness of real-time video counseling on health risks for smoking, nutrition, alcohol consumption, physical activity, and obesity. The COM-B model suggests that behavior is a result of 3 factors: capability (psychological or physical), opportunity (physical or social), and motivation (reflective or automatic) [29]. Real-time video counseling may maximize capability, opportunity, and motivation to encourage behavior change in the following ways. First, a real-time video counseling intervention for health risks of smoking, nutrition, alcohol consumption, physical activity, and obesity can assist participants in realizing their capability by enhancing their knowledge about effective behavior change methods, situations, and environments that act as triggers for their behaviors. Second, real-time video counseling may remove barriers such as the distance and time to travel to access face-to-face treatment, thereby increasing the opportunity for behavior change. Third, real-time video counseling can include motivational interviewing and cognitive behavioral therapy techniques to increase motivation to improve factors for smoking, nutrition, alcohol consumption, physical activity, and obesity [29].

One systematic review has examined the effectiveness of various technology-based interventions on smoking cessation, including real-time video counseling; however, the review only included studies of participants with low socioeconomic status or disadvantaged populations [30]. This review identified only 1 study that found no significant difference between video counseling and telephone counseling on smoking cessation [31]. A Cochrane systematic review examined the effectiveness of real-time video counseling for smoking cessation only and found limited evidence that suggested no difference between video counseling and telephone counseling [32]. However, this systematic review excluded studies that measured smoking cessation <6 months postbaseline [32]. To the best of our knowledge, there are no other systematic reviews that have examined the effectiveness of individual real-time video support for addressing each of the risk factors for smoking, nutrition, alcohol consumption, physical activity, and obesity.

### Objective

This systematic review aimed to examine the effectiveness of individual real-time video counseling on health risks for smoking, nutrition, alcohol, physical inactivity, and obesity relative to (1) a no-intervention control group or (2) other modes of intervention delivery.

## Methods

### Narrative Review

This narrative review follows the guidelines of the Preferred Reporting Items for Systematic Reviews and Meta-Analyses (PRISMA) [33] and was completed as per the protocol registered with the International Prospective Register of Systematic Reviews (PROSPERO; registration number: CRD42017071885). Meta-analyses were not undertaken because of the heterogeneity between studies (eg, clinical vs nonclinical populations) and the small number of studies examining the effectiveness of real-time video counseling for each risk factor for smoking, nutrition, alcohol consumption, physical activity, and obesity.

### Search Strategy

The electronic databases Cochrane Register of Controlled Trials (via Cochrane Library), MEDLINE (Medical Literature Analysis and Retrieval System Online; from 1946), EMBASE (Excerpta Medica dataBASE; from 1947), PsycINFO (from 1806), and Scopus were searched from inception to retrieve studies published up to November 21, 2019, that described a real-time video counseling intervention (eg, video conferencing or video consultation or telehealth or telemedicine) for modifying health risks for smoking, nutrition, alcohol consumption, physical activity, and obesity. The reference lists of included trials were also manually searched to retrieve any other relevant studies.

The database search consisted of focused text word searches and medical subject heading searches. The search terms were divided into 3 groups: (1) smoking, nutrition, alcohol, physical activity, and obesity behavior (ie, tobacco use, nutrition, alcohol drinking, physical activity, obesity, healthy lifestyle, lifestyle), (2) video communication intervention (ie, telemedicine, videoconferencing, remote consultation, Skype, Viber, webcam, Talky Core, WhatsApp, FaceTime, Messenger, Google Hangouts), and (3) study design (ie, randomized controlled trial, cluster randomized trial). Textbox 1 outlines the search strategy.

**Search strategy.**

- Nicotine/
- Tobacco/
- exp “Tobacco Use Cessation”/
- exp “Tobacco Use”/
- (Cigar* or smok* or tobacco or nicotine).tw.
- 1 or 2 or 3 or 4 or 5
- exp Healthy Lifestyle/
- exp Life Style/
- (lifestyle* or life style*).tw.
- nutrition*.mp.
- exp Fruit/
- exp Vegetables/
- (fruit* or vegetable*).tw.
- 7 or 8 or 9 or 10 or 11 or 12 or 13
- exp Alcohol Drinking/
- exp Alcoholism/ or exp Drinking Behavior/
- exp Alcoholic Intoxication/
- (Alcohol* or drinking).tw.
- 15 or 16 or 17 or 18
- exp Exercise/
- physical activity.mp.
- exp Sedentary Lifestyle/
- (physical activit* or physical inactivit*).tw.
- (exercise* or Sport*).tw.
- 20 or 21 or 22 or 23 or 24
- exp Overweight/
- Obes*.tw.
- 26 or 27
- exp Telemedicine/
- exp Videoconferencing/
- Remote Consultation/
- (skype or viber or webcam or talky core or whatsapp or facetime or messenger or google* hangouts).mp. (mp=title, abstract, original title, name of substance word, subject heading word, keyword heading word, protocol supplementary concept word, rare disease supplementary concept word, unique identifier, synonyms)
- ((real time or realtime) adj3 (counsel* or support* or therap* or conference or consult*)).tw.
- (remote adj3 (communicat* or consult*)).tw.
- 29 or 30 or 31 or 32 or 33 or 34
- 6 or 14 or 19 or 25 or 28
- 35 and 36
- exp Randomized Controlled Trial/
- exp Randomized Controlled Trials as Topic/
- exp Clinical Trial/
- exp Clinical Trials as Topic/
- exp Random Allocation/
- Random*.tw.
- Trial.tw.
- 38 or 39 or 40 or 41 or 42 or 43 or 44
- 37 and 45

### Eligibility Criteria

Studies were included in this review if they met the following criteria:

1. Study design: randomized trials or cluster randomized trials. Randomized trials and cluster randomized trials were included because these designs are considered the gold standard for measuring effectiveness [34].
2. Study participants: any population (ie, general population, patients).
3. Setting: any setting, including community and health care settings.
4. Intervention: video communication was used as the mode to deliver individual, one-on-one support (ie, Skype, FaceTime, Facebook Messenger, WhatsApp, or any preferred individual real-time video communication platform).
5. Comparators: the comparators included a no-intervention control group or any other form of support to address the risk factors for smoking, nutrition, alcohol consumption, physical activity, and obesity, such as written materials, telephone counseling, web-based support, and face-to-face interventions.
6. Language: studies published in English.
7. Outcome measures: any measure of an individual’s smoking (eg, smoking cessation, quit attempts), nutrition (eg, serves of fruit and/or vegetables, calories), alcohol (eg, number of standard drinks of alcohol), physical activity (eg, number of minutes of moderate or vigorous physical activity or metabolic equivalent [MET] minutes), or obesity (eg, BMI, waist circumference).

### Study Selection

After removing duplicate records, 2 authors (JB and FT, PA, or MM) independently screened the titles and abstracts of all records using either EndNote or Covidence. Papers that did not meet the eligibility criteria were excluded. Two reviewers independently examined the full text of the papers that were deemed eligible or whose eligibility was uncertain based on the title and abstract screening. Two reviewers met and discussed any discrepancies until a consensus was reached. The reasons for exclusion were recorded for all full text papers assessed that were ineligible.

### Data Extraction of Study Characteristics

Two authors (JB and AB or EB) independently extracted the following data from eligible studies: authors and country, years data collected, study design, sample characteristics, recruitment method, eligibility criteria, participation rate, treatment conditions, the video intervention received, retention at follow-up, outcome measures, the comparators, and costs. All discrepancies were resolved between the 2 reviewers through discussion, and a third reviewer (FT) was consulted when necessary.

### Methodological Quality Assessment

The quality assessment of each included study was assessed independently by 2 reviewers (JB and FT). The Quality Assessment Tool for Quantitative Studies developed by the Effective Public Health Practice Project was used to assess methodological quality [35] according to the instructions described in the Quality Assessment Tool for Quantitative Studies Dictionary [36]. The Quality Assessment Tool for Quantitative Studies assesses randomized and nonrandomized trials in relation to 6 components: selection bias, study design, confounders, blinding, data collection methods, withdrawals, and dropouts. Each study was rated as *strong*, *moderate*, or *weak* on each of these components. An overall global rating was then assigned to each study, with studies classified as *strong* (no weak ratings), *moderate* (1 weak rating), or *weak* (2 weak ratings).

## Results

**Figure 1:**
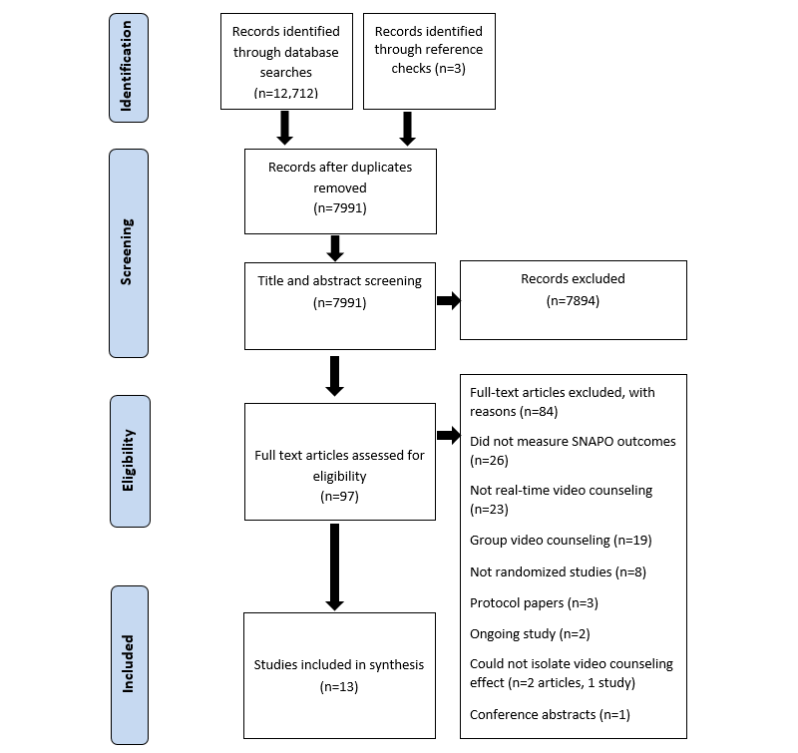
PRISMA (Preferred Reporting Items for Systematic Reviews and Meta-Analyses) diagram of the screening and selection process. SNAPO: smoking, nutrition, alcohol consumption, physical activity, and obesity.

After removing duplicates, a total of 7991 records were screened. Of these, 7894 records were excluded at the title and abstract screening stage, and 97 full text records were assessed for eligibility (Figure 1). A total of 84 of the 97 full text records were excluded for the following reasons: 26 did not measure smoking, nutrition, alcohol consumption, physical activity, and obesity outcomes; 23 did not use any form of video counseling intervention; 19 involved group video counseling and not individual video counseling [37-54]; 3 were protocol papers [55-57]; 8 were not randomized studies [58-65]; 2 studies were ongoing [32,66]; 1 was a conference abstract [67]; and 2 studies described a multicomponent intervention, and it was not possible to isolate the effect of real-time video counseling [68,69]. The remaining 13 eligible studies were included in the review. Figure 1 presents the PRISMA diagram for screening and selection.

### Study Characteristics

Four studies focused on individual video counseling for smoking cessation [31,70-72], 3 studies focused on alcohol consumption [73-75], 3 studies focused on physical activity [76-78], and 3 studies focused on obesity [79-81]. No trial examined the effectiveness of real-time video counseling on nutrition. Most trials were funded by grants from the government [76,77,79-81] or university [71,73]. One study reported receiving no funding [75], 1 study was funded by the Craig H. Neilsen Foundation [78], and 1 study was funded by CureApp Inc [72]. All 13 studies were published from 2010 onward.

#### Smoking Cessation

Three randomized trials that examined the effectiveness of real-time video counseling compared with telephone counseling for smoking cessation were conducted in the United States [31,70,71]. One trial conducted in Japan compared real-time video counseling with face-to-face counseling [72]. The studies followed-up participants for 3 [70], 6 [71,72], or 12 months [31]. One trial focused on a clinical population of women with HIV [71], whereas 3 studies included nonclinical populations [31,70,72]. The sample sizes were 49 [70,71], 115 [72], and 566 participants [31]. Two studies recruited women only [70,71], whereas another study had a majority of female participants (65%) [31] and 1 study had a majority of male participants (81%) [72]. Across the 4 trials, the mean age was 45 (SD 11.7) to 55 years (SD 11) [31,70-72]. The participation rates were 36.66% (566/1544) [31], 52% (49/94) [71], 64% (49/77) [70], and unclear for 1 study [72]. The retention rates at follow-up were 87.6% (496/566) at 12 months [31], 55% (27/49) [71] and 97.4% (112/115) [72] at 6 months, and 78% (38/49) at 3 months [70].

Multimedia Appendix 1 [31,70-72] provides a detailed description of the study characteristics. The trial in rural United States (Kansas) recruited participants from 20 primary care clinics and through community-based activities (eg, radio interviews, health fairs, and religious organizations) [31], whereas another trial recruited Korean-American women through web-based communities and newspaper advertisements [70]. The US trial among women living with HIV had participants referred by health workers, professional health networks, advertisements on free websites, and Craiglist [71], whereas Nomura et al [72] recruited participants from community clinics or centers.

One trial delivered the intervention through 4 individually tailored sessions at the clinic [31], whereas participants were offered 5 internet-based video counseling calls in another study [72]. In 2 studies, participants received up to 8 individual video counseling calls at home [70,71]. Participants from the rural US study also received written materials on smoking cessation and pharmacotherapy [31], whereas studies with Korean-American women and women living with HIV offered nicotine patches to their participants [70,71]. Participants were followed-up at 3, 6, and 12 months for 1 study [31] and at the end of the intervention and at 3 and 6 months for another study [71]. In the study with Korean-American women, participants were followed-up for 3 months [70], and in the study conducted in Japan, participants were followed-up at 3 and 6 months [72].

Three studies reported prolonged abstinence, 1 at 3 months [70], 1 at 6 months [71], and 1 at 12 months [31]. One study reported continuous abstinence between 9 and 12 weeks and 9 and 24 weeks [72]. Three studies reported 7-day point prevalence abstinence outcomes at 1, 2, and 3 months [70]; at the end of the intervention; at 3 and 6 months [71]; and at 12 months [31]. Only the rural US study reported provider costs for real-time video counseling and telephone counseling interventions [31].

#### Alcohol Use

Multimedia Appendix 2 [73-75] provides a detailed description of the study characteristics. One study was conducted in Denmark [73] and the other 2 studies were conducted in the United States [74,75]. Participants were recruited from public outpatient alcohol clinics [73], a web-based research participation system for undergraduate students [75], and community supervision offices for people with substance abuse [74]. One study included a clinical population with an alcohol dependence syndrome [73], whereas the other 2 studies were conducted with nonclinical populations [74,75]. The sample sizes were 51 [75], 71 [73], and 127 [74] across the studies. The proportion of men was high in 2 studies, specifically 73% (52/71) [73] and 81.1% (103/127) [74], whereas the majority were women (60.8%) in 1 study [75]. The mean age was 19 (SD not reported) [75] and 47 years (SD 12.8) [73], with a median age of 30.5 years [74]. The participation rate was 63% [73] and 73% [74] across 2 studies and unclear in 1 study [75]. One trial compared video counseling (telehealth) with face-to-face treatment only [75]. The other 2 trials compared individual video conferencing plus face-to-face support (treatment as usual) with face-to-face only support (treatment as usual) [73,74].

In 1 study, participants received up to 5 sessions with the therapist via videoconference and were followed-up at 3 months [74], whereas another study offered between 1 and 3 sessions a week at the initial stages, followed by 1 session every other week for about 7 months, and participants were followed-up until 12 months [73]. In the third study, participants received only 2 sessions and were followed-up at 1, 2, and 3 months [75].

Alcohol consumption was measured at 3, 6, and 12 months in 1 study [73], at 3 months in another study [74], and at 1, 2, and 3 months in the third study [75]. The costs of the video intervention and face-to-face support (treatment as usual) were not reported in any of these 3 studies [73-75].

#### Physical Activity

Three trials examined the effectiveness of individual video counseling to increase physical activity. The trials were conducted in Australia [76], the United States [77], and Canada [78]. Participants were recruited from print and web advertising in 1 study [76], whereas in the other 2 studies, participants were recruited from their primary care provider [77] or from outpatient rehabilitation hospitals in Montreal, a local adapted fitness center, an organization representing persons with spinal cord injury (SCI), pre-existing databases of previous research participants, and social media platforms [78]. Two physical activity trials were conducted with clinical populations, specifically people with paraplegia [78] and people with diabetes [77], whereas 1 study was conducted with a nonclinical population [76]. The sample sizes across the 3 studies were 24 [78], 154 [76], and 1650 participants [77]. In 2 trials, most participants were female (117/154, 76.0% [76] and 1037/1650, 62.84% [77]), whereas in the third study, the majority were male 73% (16/24) [78]. The average BMI was 31 kg/m^2^ [76] and 32 kg/m^2^ [77] in 2 studies, and BMI was not reported in the Canadian study [78]. Across the studies, the mean age ranged from 51.64 (SD 12.3) [78] to 70.9 (SD 6.63) years [77]. One trial compared real-time video counseling plus computer-tailored advice with computer-tailored web-based physical activity intervention only (advice tailored to an individual with graphs and text) and to a waitlist control group [76]. The intervention participants in this trial received tailored physical activity advice plus video counseling every 2 weeks for 8 weeks (“My Activity Coach”) [76]. These were four 10-min coaching sessions with a behavioral expert using a web-based video-calling program (Skype) compared with computer-tailored physical activity advice only and a waitlist control. Participants were followed-up at week 9 and at 6 months [76]. In the second study [77], the intervention comprised video counseling (home video calls) with a diabetes educator conducted every 4 to 6 weeks for self-management, which was compared with face-to-face care (usual clinic-based care), and participants were followed-up for 5 years [77]. The third trial compared video counseling (intervention) versus regular routine (control) in adults with SCI in Canada [78]. The intervention comprised 1 leisure time physical activity (LTPA) counseling session per week for 8 weeks, resulting in a total of 8 counseling sessions [78]. Participants were then followed-up at weeks 6 and 10 [78].

One trial assessed physical activity in minutes per week at week 9 and 6 months [76], another assessed the rate of decline in physical activity in older participants over a 5-year period [77], and the third trial assessed total LTPA at 6 and 10 weeks [78]. One trial reported a retention rate of 92% at 10 weeks [78], and 2 trials reported retention rates of <50% [76,77]. No cost information was provided for any of the studies. Multimedia Appendix 3 [76-78] provides a detailed description of the study characteristics.

#### Obesity

Multimedia Appendix 4 [79-81] provides a description of the study characteristics examining the effectiveness of video counseling on obesity. Three trials [79-81] used individually delivered real-time video counseling to target obesity. The trials targeted clinical populations with lifestyle conditions such as diabetes [79,80], hypertension [80], and overweight or obesity (BMI>25 kg/m^2^) [79-81]. One study was conducted in Japan [80], another study in Denmark [79], and the third in the United States [81]. In 1 study, the participants were recruited through telephone calls from outpatient departments [79] and in another study via community advertisements [80], whereas it was unclear how participants were recruited in the third study [81]. The sample sizes were 30 [81], 68 [80], and 165 [79]. The mean age was 66 years (SD 1.7) [80] and 58 years (SD 9.3) [79] in 2 studies and ranged from 42.2 to 44.5 years across the 3 groups in the third study [81]. The majority of participants were male 63.8% (106/166) in 1 trial [79] and female 65% (44/68) in another trial [80], whereas the gender distribution was not reported in the third trial [81]. The trials reported BMI and physical activity outcomes at 3 months [80], BMI and waist to hip ratio at 8 and 14 months [79], and physical activity and body weight loss over 12 weeks [81].

The video counseling intervention was compared with either individualized documented reports (individualized written reports at 3 time points addressing lifestyle modifications) [80] or usual care (face-to-face) [79] or face-to-face or a control group that received no feedback from mobile health devices and no health coaching sessions [81]. In 1 study, real-time video consultations were delivered 3 times in 3 months [80]. In another study, they used video add-ons in usual clinic-based care (every 3-6 months) with a health care center nurse via a tablet for 32 weeks [79]. In the third study, the video counseling intervention participants received health coaching educational materials and weekly individualized videoconferencing by a multidisciplinary team (registered dietitian, exercise physiologist, and medical doctor) based on data uploaded over the 12-week intervention [81]. None of the trials provided any information on the cost of the interventions.

### Effectiveness of Real-Time Video Counseling on Smoking Cessation

In the nonclinical populations [31,70,72], Richter et al found no significant difference between video counseling and telephone counseling for self-reported 7-day point prevalence abstinence at 3 months and 6 months and reported no significant difference between video counseling (9.8%) and telephone counseling (12%) in biochemically verified 7-day point prevalence abstinence and prolonged abstinence (video 8.1% and telephone 7.6%) at 12 months [31]. Kim et al [70] reported no significant difference between biochemically validated 7-day point prevalence abstinence in the video counseling arm (33.3%) compared with the telephone counseling arm (28%) at 3 months. Prolonged abstinence also did not differ significantly between the video counseling arm (29.2%) and the telephone counseling arm (28%) [70]. Nomura et al [72] found no significant difference between video counseling and face-to-face sessions for biochemically validated continuous abstinence rate between weeks 9 to 12 (video 81.0%; face-to-face 78.9%) and weeks 9 to 24 (video 74.1%; face-to-face 71.9%).

In a study conducted with women living with HIV, a clinical population, the video counseling group was significantly more likely than the telephone counseling group to achieve biochemically verified point prevalence abstinence at 3 months (video 33.3%; telephone 4.8%) and 6 months postquitting (video 38.1%; telephone 4.8%) [71]. This study also found that those in the video counseling group were significantly more likely than the telephone counseling group to achieve a 6-month prolonged abstinence (video 33.3%; telephone 4.8%) [71].

### Effectiveness of Real-Time Video Counseling on Alcohol Use

Two studies were conducted in a nonclinical population [74,75]. In 1 study [74], compared with usual care (social service clinician) only, the real-time video communication group did not significantly differ on any alcohol consumption, days of drinking, drinks per week, and days experiencing alcohol problems at 3 months. In the second study, there was no significant difference in the change in Alcohol Use Disorders Identification Test scores between the video support group and the face-to-face support group from baseline to 1 month posttreatment and 1 to 3 months posttreatment [75]. Similarly, there was no significant difference in Rutgers Alcohol Problem Index (RAPI) scores between the groups at 1-month follow-up and the decrease in RAPI scores from baseline to 1 month and between 1- and 3-month follow-ups [75].

Only a single study was conducted in a clinical population (alcohol dependent) [73]. Tarp et al [73] found no significant difference between video counseling options and usual face-to-face care in the change from baseline to 12 months in the number of days of alcohol consumption in the past month and days of excessive alcohol consumption in the past month.

### Effectiveness of Real-Time Video Counseling on Physical Activity

One study for physical activity was conducted among a nonclinical population [76]. In this study, there was a significant change in physical activity (minutes per week) from baseline to week 9 between the tailoring and video-coaching intervention for physical activity and the control group, but there was no significant change between the tailoring and video-coaching intervention and the tailoring-only intervention [76]. From baseline to 6 months, the change in physical activity (minutes per week) did not significantly differ between the tailoring plus video-coaching intervention and either of the other groups [76].

Two of the physical activity studies were conducted among a clinical population [77,78].

In the study by Weinstock et al [77] among people with diabetes, there was a significantly lower rate of decline in physical activity over time in the video counseling group than in the usual care group. In the second study among people with paraplegia, Chemtob et al found that compared with the control group, the video counseling group reported greater total minutes of LTPA at 6 weeks (Hedge g=0.87) and 10 weeks (Hedge g=0.85) [78]. For moderate and vigorous physical activity, moderate effect sizes were found at 6 weeks (Hedge g=0.52) and small effect sizes were found at 10 weeks (Hedge g=0.34) favoring the video counseling group over the control group [78].

### Effectiveness of Real-Time Video Counseling on Obesity

All studies examining the effectiveness of real-time video counseling on obesity were conducted with clinical populations [79-81]. One study compared video counseling with usual care and found no changes in BMI or waist to hip ratio [79]. The second study found a significant change in BMI from preintervention to postintervention (3 months) between the video counseling intervention and the individualized monthly document reports group but no significant change between the groups in average steps per day from preintervention to postintervention [80]. The third study by Johnson et al [81] found that the video counseling group achieved significantly greater weight loss from baseline to 12 weeks than the in-person group and the control group. This study also reported that the video counseling group had significantly higher steps per day than the in-person group at week 4 and the control group at weeks 6, 8, 9, and 11 [81].

### Satisfaction With Real-Time Video Counseling for Smoking Cessation

Two studies [31,71] compared the satisfaction of real-time video counseling for smoking cessation with telephone counseling. In 1 study with a nonclinical population [31], those in the video counseling group (97%) were significantly more likely to recommend the program to family and friends than those in the telephone counseling arm (91.9%), but no between-group differences were found for other satisfaction measures. In the other study with a clinical population, there was no significant difference in mean satisfaction scores between the video counseling and the telephone counseling groups [71].

### Satisfaction With Real-Time Video Counseling for Alcohol Use

Of the 3 [73-75] studies on alcohol consumption, 1 study [75] assessed satisfaction. This study compared treatment satisfaction between the video counseling group and the face-to-face support group in a nonclinical population and found no significant difference between the 2 groups for client satisfaction questionnaire scores at either session 1 or session 2 [75].

### Satisfaction With Real-Time Video Counseling for Physical Activity

One study on physical activity in a nonclinical population [76] reported on the satisfaction between tailoring and video coaching compared with tailoring-only. Alley et al [76] found no significant difference between these groups on program satisfaction scores.

### Satisfaction With Real-Time Video Counseling for Obesity

All 3 studies conducted with clinical populations [79-81] that focused on obesity did not assess satisfaction with video counseling compared with the comparator used.

### Methodological Quality Assessment for Real-Time Video Counseling Studies

Table 1 outlines the methodological quality ratings for each study across the 6 components (selection bias, study design, confounders, blinding, data collection methods, withdrawals, and dropouts) and the overall global rating. In terms of the global rating, only 1 study was rated as moderate (a physical activity trial) [77], whereas 12 studies had a weak global rating (4 smoking cessation trials [31,70-72], 3 alcohol trials [73-75], 2 physical activity trials [76,78], and 3 obesity trials [79-81]).

**Table 1:** Methodological quality assessment of eligible studies.

Study		Selection bias		Study design		Confounders		Blinding		Data collection methods		Withdrawals and dropouts		Global rating	
**Smoking**															
	Kim et al [70]		Weak		Strong		Weak		Weak		Strong		Moderate		Weak
	Kim et al [71]		Weak		Strong		Weak		Weak		Strong		Weak		Weak
	Nomura et al [72]		Weak		Strong		Strong		Weak		Strong		Strong		Weak
	Richter et al [31]		Weak		Strong		Strong		Weak		Strong		Strong		Weak
**Alcohol**															
	King et al [75]		Weak		Strong		Moderate		Weak		Strong		Weak		Weak
	Staton-Tindall et al [74]		Moderate		Strong		Weak		Weak		Weak		Strong		Weak
	Tarp et al [73]		Weak		Strong		Strong		Weak		Strong		Moderate		Weak
**Physical activity**															
	Alley et al [76]		Weak		Strong		Weak		Weak		Strong		Weak		Weak
	Chemtob et al [78]		Weak		Strong		Weak		Moderate		Strong		Strong		Weak
	Weinstock et al [77]		Weak		Strong		Strong		Moderate		Moderate		Strong		Moderate
**Obesity**															
	Hansen et al [79]		Weak		Strong		Strong		Weak		Strong		Strong		Weak
	Homma et al [80]		Weak		Strong		Weak		Weak		Strong		Strong		Weak
	Johnson et al [81]		Weak		Strong		Strong		Weak		Strong		Strong		Weak

Most studies (n=12) were rated as weak for selection bias because the participation rate was <60% or unclear [31,70-73,75-81]. Blinding of either the outcome assessor or the participant was also another component where most (n=11) studies were rated as weak [31,70-76,79-81]. Regarding confounders, 6 studies had a weak rating because <60% of the potential confounders were controlled for [80] or it was unclear whether potential confounders were controlled for [70,71,74,76,78]. Data collection was only weak in a single study [74] because it was unclear whether the tools used were reliable. Three studies were rated as weak in relation to withdrawals and dropouts because they reported low overall retention rates of 30% [75] and 38% [76] or low retention for one arm of the study (48%) [71].

## Discussion

### Principal Findings

This is the first review to examine the effectiveness of individual real-time video counseling on smoking, nutrition, alcohol consumption, physical activity, and obesity. This review focused on real-time video communication technology, an emerging intervention delivery mode. The overall results suggest that video counseling is neither more nor less effective in modifying smoking and alcohol consumption but may have particular benefits for addressing physical inactivity and obesity. Given that the effectiveness of video counseling was similar to conventional methods used to treat smoking and alcohol consumption and that many individuals with nicotine dependence or alcohol dependence may not join and complete conventional treatment [82,83], video counseling provides another option to engage people with nicotine dependence or alcohol dependence who are unlikely to use conventional treatment or drop out of such support. If real-time video counseling is at least equally effective to existing treatments such as face-to-face interventions, then the smoking, nutrition, alcohol consumption, physical activity, and obesity program providers should consider including video counseling as an additional option into their services. The importance of a variety of delivery modes has been demonstrated during the COVID-19 pandemic, where access to face-to-face services has been restricted, whereas in contrast, real-time video counseling for risks for smoking, nutrition, alcohol consumption, physical activity, and obesity is sustainable in this context. The cost of video counseling compared with other modes of delivery is difficult to determine because it was reported in only 1 smoking cessation study [31], which required participants to travel to the clinic to receive video sessions (instead of receiving video sessions at home).

Of the 4 studies that examined the effectiveness of video counseling on smoking cessation [31,70-72], only 1 study reported a significant difference between video counseling and telephone counseling at the 3- and 6-month follow-up, which favored the video counseling group [71]. All studies that focused on smoking cessation were comparative effectiveness trials, and there is currently no evidence available on the effectiveness of real-time video counseling compared with a no-intervention or minimal support (eg, written self-help materials) control group for smoking cessation. The global methodological quality rating of the 4 studies that assessed the effectiveness of real-time video counseling for smoking cessation was weak, suggesting that the methodological rigor of the evidence needs to be improved, particularly in relation to blinding. However, given the nature of trials that examine the effectiveness of real-time video counseling, blinding would be difficult [84]. Additionally, 3 of the 4 studies were conducted in specific populations, such as Korean-American women [70], women living with HIV [71], and rural smokers [31], hence limiting the generalizability of the findings. Given that quitlines provide telephone counseling as part of their standard practices [85,86], and 2 studies report no differences between telephone counseling and video counseling for smoking cessation [31,70] whereas 1 study suggests that video counseling for smoking cessation is superior to telephone counseling [71], quitline providers could consider expanding their routine services to include real-time video counseling.

The evidence in 3 studies indicated that there was no significant difference between real-time video counseling and face-to-face counseling (usual care) for reducing alcohol consumption [73-75]. All studies had a weak global rating with small sample sizes and low retention rates, which resulted in limited power to detect any differences. Moreover, one of the studies included a largely white population (98%) [74] and therefore may have limited generalizability with respect to other cultures and populations, such as those in low- and middle-income countries. Nonetheless, given that real-time video counseling overcomes barriers associated with face-to-face treatment for alcohol consumption such as time and distance [87], and no differences were found between face-to-face treatment (usual care) and video consultations [73-75], service providers could consider offering real-time video counseling as an additional option for modifying alcohol consumption.

Real-time video counseling was found to significantly increase physical activity when compared with usual care at week 9 [76] and after 5 years [77]. However, given the limitations in methodological quality and the paucity of research in this field, further randomized trials examining the effectiveness of real-time video counseling on physical activity are warranted. Given that the existing studies focused on high-income countries, included only obese or diabetic populations, and included participants who were predominantly women, white, and highly educated, the generalizability of the findings to other populations may be limited. Despite the limited evidence, the existing research suggests that real-time video counseling is more effective than usual care for improving physical activity. Therefore, physical activity service providers could consider offering real-time video counseling as part of their routine practice.

Two studies that focused on obesity reported a significant change in BMI from preintervention to 3 months between the video counseling intervention and the individualized monthly document reports group [80], and the video counseling group achieved significantly greater weight loss from baseline to 12 weeks than the in-person group and control group [81]. Only 1 study found no changes in BMI and waist to hip ratio between the video add-on group and the face-to-face treatment group (usual clinic-based care) [79]. Two of the 3 studies that focused on obesity also reported physical activity outcomes. There was a significant difference for 1 study reporting on increasing steps per day for video counseling compared with the control group that favored the video counseling group [81], whereas 1 study found no difference in change in average steps per day between video counseling and individualized documented reports [80]. All 3 studies were rated as weak for selection bias and therefore were unlikely to be representative of the target population. There is some evidence to suggest that real-time video counseling is effective for obesity. Further randomized trials assessing the effectiveness of real-time video counseling with robust methodological quality on obesity are required.

It is worth noting that 7 studies [71,73,77-81] have focused on clinical populations (1 HIV [smoking trial] [71], 3 diabetes [1 physical activity and 2 obesity trials] [77,79,80], 1 alcohol dependence syndrome [alcohol trial] [73], 1 paraplegia [physical activity] [78], and 3 obesity [obesity trials] [79-81]). Four of these studies in clinical populations found that video counseling was superior to the comparator [71,77,80,81], whereas video counseling was as effective as the comparator in 3 trials [73,78,79]. Six studies [31,70,72,74-76] focused on nonclinical populations. Five of the studies with nonclinical populations reported video counseling to be as effective as the comparator group [31,70,72,74,75], whereas video counseling was superior to the comparator in 1 study in the short term but not in the longer term [76]. To expand the evidence, future research is needed to examine the effectiveness of real-time video counseling for smoking, nutrition, alcohol consumption, physical activity, and obesity behaviors in both clinical and nonclinical populations.

Four studies (1 smoking [31], 1 alcohol [74], and 2 physical activity [76,77]) were either conducted exclusively in rural areas or rural and/or regional areas were targeted along with urban locations as part of recruitment. One study with rural residents reported no difference between video counseling and telephone counseling for smoking cessation [31] and another study found no difference between video counseling and face-to-face support on alcohol consumption [74]. In studies that targeted rural and/or regional areas along with urban locations, real-time video counseling was found to significantly increase physical activity compared with usual care at week 9 [76] and after 5 years [77]. Given that rural populations may face challenges accessing services because of distance, real-time video counseling, which is either as effective or more effective than control or comparator interventions, may overcome barriers to accessing smoking, nutrition, alcohol consumption, physical activity, and obesity services in rural locations.

Satisfaction with video counseling was compared with a comparator group in 2 smoking trials [31,71], 1 alcohol trial [75], and 1 physical activity trial [76]. Three of these 4 studies reported no significant differences between real-time video counseling and the comparator group in terms of satisfaction [71,75,76], whereas 1 smoking cessation trial reported that those in the video counseling group were more likely to recommend the program to family and friends than those in the telephone counseling arm [31]. Overall, these results suggest that in terms of satisfaction, those offered real-time video counseling to address smoking, alcohol consumption, and physical activity risks are at least as satisfied with this program as those offered other conventional methods. This provides further support for the potential of real-time video counseling to be integrated into existing preventive care programs.

### Limitations

Although a comprehensive search strategy was conducted, the studies included were disproportionate across smoking, nutrition, alcohol consumption, physical activity, and obesity outcomes. Namely, there was no intervention targeting nutrition and only 4 studies targeting smoking, 3 studies targeting alcohol consumption, 3 studies targeting physical activity, and 3 studies targeting obesity. The lack of studies limits the conclusions that can be made and highlights the need for more trials assessing the effectiveness of individual, real-time video counseling that target these behaviors. Additionally, some studies that were not published in a peer-reviewed journal or not written in English were excluded, and some studies may have been missed through limitations in the searched databases [88]. Another limitation is that all included studies were conducted in high-income countries; therefore, the findings may not be generalizable to populations in developing countries or those with a diverse socioeconomic status and cultural background. Furthermore, more than half of the studies had a sample size of <100 participants [70,71,73,75,78,80,81], which may have resulted in inadequate statistical power to detect differences between groups. Additionally, in terms of methodological quality, 12 of the 13 studies had a global rating of weak, with improvements needed particularly for selection bias and blinding. Furthermore, the quality assessment for each study was based on the information reported in the publication by the authors [36], which may have had an impact on quality assessment.

This review highlights the need for more research trials examining the effectiveness of video counseling for health risks for smoking, nutrition, alcohol consumption, physical activity, and obesity. Future research should assess the effectiveness of video counseling for each health risk behavior in various populations (eg, general population, high-risk groups, and minority groups), settings (eg, health care settings, community settings, rural and remote locations), countries (eg, low- and middle-income), and cultures (eg, culturally and linguistically diverse groups, indigenous) to build upon the evidence-base and improve the generalizability of the findings. Studies examining the effectiveness of real-time video counseling for health factors of smoking, nutrition, alcohol consumption, physical activity, and obesity should consider having a larger sample size to increase the power to detect differences between groups, include populations with diverse socioeconomic and cultural backgrounds, and reduce selection bias through random selection and blind assessors and participants where possible. Future research could also examine the effectiveness of real-time video counseling for other behaviors such as sleep, health care seeking behaviors, adherence to treatments, and mental health.

Such evidence is important for informing the practices of public health prevention programs and health practitioners. Real-time video consultations have been successfully used by health practitioners for various patient-clinician consultations of long-term conditions such as heart failure, depression, schizophrenia, stroke, asthma, spinal cord injury, and chronic pain [89]. Similarly, this review suggests that health practitioners could extend the use of real-time video consultations to address the risks of smoking, nutrition, alcohol consumption, physical activity, and obesity with clients. Public health programs such as quitlines and other telephone or face-to-face services that aim to modify the risks of smoking, nutrition, alcohol consumption, physical activity, and obesity could also consider including the option for clients to choose to have support delivered via real-time video consultations. The choice to utilize real-time videoconferencing may be influenced by many factors such as client preference and funding available to providers and conditions of such funding. The use of video communication technology to provide health care services during the COVID-19 pandemic illustrates the sustainability of real-time video counseling for the risks of smoking, nutrition, alcohol consumption, physical activity, and obesity and the accessibility and reach of this intervention.

### Conclusions

This review focused on effectiveness, costs, and satisfaction, factors that contribute to decision making regarding the mode by which care is delivered to clients. Policy makers and service providers also take into account other factors when making a decision about whether to integrate an intervention into their routine practices, such as feasibility, each from a provider and a client perspective. Further research is required to determine the relative benefits of video counseling in terms of these other policy and practice decision-making factors.

## Appendix

Multimedia Appendix 1 Characteristics of studies examining the effectiveness of video counseling on smoking cessation .

Multimedia Appendix 2 Characteristics of studies examining the effectiveness of video counseling on alcohol consumption.

Multimedia Appendix 3 Characteristics of studies examining the effectiveness of video counseling on physical activity.

Multimedia Appendix 4 Characteristics of studies examining the effectiveness of video counseling on obesity.

## Abbreviations

COM-B: capability, opportunity, motivation, and behavior
DALY: disability-adjusted life year
LTPA: leisure time physical activity
MET: metabolic equivalent
PRISMA: Preferred Reporting Items for Systematic Reviews and Meta-Analyses
PROSPERO: Prospective Register of Systematic Reviews
RAPI: Rutgers Alcohol Problem Index
SCI: spinal cord injury
