# Supplementary material for: Effectiveness of Individual Real-Time Video Counseling on Smoking, Nutrition, Alcohol, Physical Activity, and Obesity Health Risks: Systematic Review
Source: J Med Internet Res. 2020 Sep 11;22(9):e18621. doi: 10.2196/18621 (PMC7519427; doi:10.2196/18621)
Supplement: Multimedia Appendix 1 [file jmir_v22i9e18621_app1.docx]

**Table 1.** Characteristics of studies examining the effectiveness of video counseling on smoking cessation.

| Author; country; years data collected; source of funding | Study design; setting | Population; sample characteristics | Recruitment method; eligibility criteria; participation rate; retention at follow-up | | Video intervention mode; video intervention description; video intervention received | Comparator mode; comparator description; comparator received | | Satisfaction measures | Outcome measures; cost |
| --- | --- | --- | --- | --- | --- | --- | --- | --- | --- |
| Kim et al [71]; USA; 2014–2015; National Institute on Drug Abuse | 2-arm randomized trial with video counseling vs telephone counseling; Korean-American women residing in communities of Boston, Centreville, Los Angeles, New York City, San Francisco, Seattle | Nonclinical n=49; Telephone n=25; mean age 44.9 years; 64% employed; 48% married; 52% completed elementary to high school; mean cigarettes per day=17.2; Video: n=24; mean age 45.3 years; 88% employed; 50% Baccalaureate/higher degree; 54.2% married/with partner; mean cigarettes per day=15.2 | Web-based communities for Korean Americans, offline newspapers; women of Korean ethnicity, aged between 18 and 65 years, smoked at least 10 cigarettes/day for the past ≥6 months, willing to quit smoking within the next 4 weeks from the baseline assessment, had no contraindication to nicotine patches, not pregnant or lactating, access to a mobile phone or computer for video calls; 64% participation rate; 77.6% retention at 3 months | Video calls via video call app; 8 weekly, 30-min sessions of individualized video counseling, nicotine replacement patches and self-help materials; mean video calls received: 5.75 (SD 3.14), 38% completed 8 sessions | | Telephone; 8 weekly, 30-min telephone smoking cessation counseling sessions and nicotine patches for 8 weeks and self-help materials; mean telephone calls received 6.72 (SD 2.19), 48% completed 8 sessions | Not stated | | 7-day point prevalence abstinence at each follow-up. 1 month (self-report): 66.7% video, 48% telephone (NS^a^). 2 months (self-report): 58.3% video, 52% telephone (NS^a^). 3 months (self-report): 41.7% video, 40% telephone (NS^a^). 3 months (salivary cotinine test): 33.3% video, 28% telephone (NS^a^). 3-month prolonged abstinence (cotinine confirmed): 29.2% video, 28.0% telephone (NS^a^); not stated |
| Kim et al [72]; USA; 2016–2018; Joseph P. Healey Research Grant and University of Massachusetts Boston – Dana Farber Harvard Cancer Centre U54 Partnership Grant | 2-arm randomized trial with video counseling vs telephone counseling; women living with HIV in the states of Massachusetts and New York | Nonclinical; n=49, 74% Black, mean age: 51.12 years, mean years living with HIV: 18.6, 30.95% married or living with partner, 57.1% have 12 years of education, 80.5% employed, mean number of cigarettes per day: 14.2 | Participants referred by health workers, professional networks of health care providers who were working with people living with HIV, advertisements on free websites, Craiglist; women, English-speaking, HIV-positive serostatus and CD4 cell count and viral load, 18–75 years old, smokers reporting at least 5 cigarettes a day for the past 6 months, access to video calling via smartphone, willingness to set a quit date within 4 weeks from the first counseling session and the usage of an approved form of birth control during the study period; 88% participation rate; 55% retention rate at 6 months after quitting | Video calls via a video call app; 8 weekly individualized video counseling sessions of smoking cessation with nicotine replacement patches for 8 weeks, HIV-tailored smoking cessation intervention; 66.7% received all 8 video counseling sessions. Each session lasted 10-30 min | | Telephone calls; 8 weekly individualized telephone counseling sessions of smoking cessation with nicotine replacement patches for 8 weeks, HIV-tailored smoking cessation intervention; 59.1% received all 8 counseling sessions, each session lasted 10-30 min | Client Satisfaction Questionnaire (mean score): 29.6 video, 28.9 telephone (NS^a^) | | Point prevalence abstinence at each follow-up. End of intervention (self-report): 71.4% video, 52.4% telephone (NS^a^). 3 months (self-report): 47.6% video, 19% telephone (S^b^). 3 months (salivary cotinine test): 33.3% video, 4.8% telephone (S^b^). 6 months (salivary cotinine test): 38.1% video, 4.8% telephone (S^b^). 6-month prolonged abstinence (cotinine confirmed): 33.3% video, 4.8% telephone (S^b^); not stated |
| Nomura et al [73]; Japan; 2018-2019; CureApp Inc | 2-arm randomized, noninferiority trial with video counseling vs face-to-face counseling; 4 community clinics in Tokyo, Japan | Nonclinical; n=115, mean age: 55 years, 81% male, median BMI: 23 kg/m^2^, median cigarettes per day: 15, median years of smoking: 34, median number of quit attempts: 1 | Participants recruited from community clinic/center; nicotine dependent, have smoking history of Brinkman index >200, and have a determination to quit smoking immediately, could use a smartphone; not clear; 97.4% retention rate at 24 weeks | Internet-based video counseling system (telemedicine); 5 video counseling sessions at weeks 2, 4, 8, 12, and 24. Also used the CASC^c^ system smartphone app, which included a mobile exhaled CO checker; 98% adherence rate at weeks 2, 4, 8, 12, and 24. Duration not stated | | Face-to-face visits; 5 face-to-face standard smoking cessation support sessions at weeks 2, 4, 8, 12, and 24. Also used the CASC^c^ system smartphone app, which included a mobile exhaled CO checker; 96% adherence rate at weeks 2, 4, 8, and 24 and 95% adherence rate at week 12. Duration not stated | Not stated | | Biochemically validated CAR^d^. CAR^d^ from weeks 9-12: 81.0% video, 78.9% face-to-face (NS^a^). CAR^d^ from weeks 9-24: 74.1% video, 71.9% face-to-face (NS^a^); not stated |
| Richter et al [31]; USA; 2009–2012; National Heart, Lung, and Blood Institute | 2-arm randomized trial with video counseling vs telephone counseling; patients of 20 primary care clinics in the rural counties in the state of Kansas | Nonclinical; n=566, 83% Caucasian, mean age: 47.4 years, 43% married, 65% female, 56.8% high school education or less, 41.7% employed full time, mean cigarettes per day: 19.7 (SD 10.3), PHQ-2^e^, depression 49.9% | Participants recruited by clinic staff on site, via mailings from clinic directors, radio interviews, health fairs, community newsletters, businesses, staff tables at Latino worksites, and religious organizations; primary care practice participating in the study, ≥18 years, smoke ≥5 cigarettes per day for 1 year, smoked 25 out of past 30 days, speak English or Spanish and have a telephone; 37% participation rate; 88% retention at 12 months | ITM^f^ at physician office/clinic; 4 individually tailored sessions of clinic-based video telemedicine and written materials with information on smoking cessation and pharmacotherapy; ITM^f^ sessions completed: ≥1=79.7%, ≥2=68.5%, ≥3=55.1%, 4=32.3%. Mean number of video calls received 2.4 (SD 1.5). Duration not stated | | Telephone; 4 individually tailored sessions via home or mobile phone and written materials with information on smoking cessation and pharmacotherapy; telephone sessions completed: ≥1= 83.5%, ≥2=76.1%, ≥3=60.6%, 4=37.3%. Mean number of phone calls received 2.6 (SD 1.5). Duration not stated | Recommend program to family or friend: 97% ITM^f^, 91.9% telephone (S^b^). NS^a^ differences between groups for satisfaction with program and length of sessions | | Self-reported 7-day point prevalence abstinence at each of 3, 6, and 12 months: presented in figure and so exact percentages could not be extracted for ITM^f^ and telephone conditions. NS^a^ at each of 3, 6, and 12 months. Biochemically verified 7-day point prevalence abstinence at 12 months, ITM^f^: 9.8%, telephone: 12.0% (NS^a^). Prolonged abstinence, ITM^f^: 8.1%, telephone: 7.6% (NS^a^). Quit attempts (among those who continued to smoke at 12 months), ITM^f^: mean=4.8 (SD 6.8), telephone: mean=4.3 (SD 5.7) (NS^a^); provider perspective: at rental space rate, ITM^f^: mean cost: US $47.04, telephone: US $53.25. At physician office visit cost, ITM^f^: US $272.65/participant, telephone: US $53.25/participant |

^a^NS: no significant difference.

^b^S: significant difference.

^c^CASC: CureApp smoking cessation.

^d^CAR: continuous abstinence rate.

^e^PHQ-2: Patient Health Questionnaire-2

^f^ITM: integrated telemedicine.
