# Supplementary material for: Effectiveness of Individual Real-Time Video Counseling on Smoking, Nutrition, Alcohol, Physical Activity, and Obesity Health Risks: Systematic Review
Source: J Med Internet Res. 2020 Sep 11;22(9):e18621. doi: 10.2196/18621 (PMC7519427; doi:10.2196/18621)
Supplement: Multimedia Appendix 2 [file jmir_v22i9e18621_app2.docx]

**Table 2.** Characteristics of studies examining the effectiveness of video counseling on alcohol consumption.

| Author; country; years data collected; source of funding | Study design; setting | Population; sample characteristics | Recruitment method; eligibility criteria; participation rate; retention at follow-up | Video intervention mode; video intervention description; video intervention received | Comparator mode; comparator description; comparator received | Satisfaction measures | Outcome measures; cost |
| --- | --- | --- | --- | --- | --- | --- | --- |
| King et al [76]; USA; years data collected not stated; none | 2-arm randomized trial with video sessions vs face-to-face sessions; undergraduates from a large Mid-Western University | Nonclinical; N=51, mean age 19 years, 60.8% female | Participants signed up on the web via SONA, a web-based system coordinating and scheduling research participation at institutions; engaged in heavy episodic drinking (4 drinks for women/5 drinks for men on 1 occasion) within the last 2 weeks; not clear; 30% retention rate at 3 months | Video sessions via Polycom: Real Presence software; 2 individual video-based sessions on alcohol intake on computer in a lab space and faxed feedback of the sessions; not stated | Face-to-face sessions; face-to-face sessions of participants and therapist and feedback via paper version; not stated | No significant difference between groups for Client Satisfaction Questionnaire (treatment satisfaction) score at session 1 or session 2 (NS^a^) | AUDIT^b^ scores: NS in the change in AUDIT scores between face-to-face and video support groups from baseline to 1 month posttreatment and 1 month to 3 months posttreatment (NS). RAPI^c^ scores: NS in RAPI scores between the video support and face-to-face conditions at 1 month follow-up, the decrease in RAPI scores from baseline to 1 month, and between 1 and 3 months of follow-up (NS); not stated |
| Staton-Tindall et al [75]; USA; years not stated; National Institute on Alcohol Abuse and Alcoholism | 2-arm randomized trial with MET^d^ via telemedicine+usual care and services as usual; rural community supervision offices | Nonclinical; N=127, 81% male, 98% white, 72.4% on parole, median age 30.5 years, 49% employed full time during year before incarceration, median of 11 years of education, 100% used any alcohol in 3 months before incarceration, average use of alcohol 8.2 years, median AUDIT score 24, median of 45 days of alcohol use in past 3 months, median of 12 drinks per day, median of 3 days per week to drinking to intoxication | Community supervision offices, offenders with referral letter for substance abuse assessment; history of at least hazardous use of alcohol, as indicated by an AUDIT score of 8+. Willingness to enroll in alcohol services, referral to the social service clinician, self-report of alcohol as a primary substance of choice, self-reported active drinking during the past year before incarceration, and willingness to attend telemedicine sessions if randomized; 73% participation rate; 87% retention at 3 months | Video conferencing via Polycom PVX software on a computer at community supervision office; 5 sessions with the therapist for MET telemedicine via video conference; mean number of sessions attended was 2 (SD=1.9), 62% of participants engaged in a minimum of 1 or 2 sessions, 37.4% attended >3 sessions. Duration not stated | Face-to-face; services as usual. Participants were assessed and referrals given from the Social Service Clinician; actual services received not stated | Not stated | Any alcohol use at 3 months (compared with usual care): MET, OR=1.29 (0.55-2.99) (NS). Days of drinking at 3 months (compared with usual care): MET, AIRR^e^=1.11 (0.41-2.99) (NS). Drinks per week at 3 months (compared with usual care): MET, AIRR=1.76 (0.48-6.46) (NS). Days experiencing alcohol problems at 3 months (compared with usual care): MET, AIRR=22.8 (0.91-5.69) (NS); not stated |
| Tarp et al [74]; Denmark; 2012–2014; Inter-ministerial Project Office, the Psychiatric Research Foundation, Region of Southern Denmark, and the Faculty of Health, University of Southern Denmark | 2-arm randomized controlled trial, TAU^f^ or TAU+optional video conferencing; outpatients in public outpatient hospital alcohol clinic in Odense, Denmark | Clinical; N=71, mean age 47.3 years, 73% male, 82% higher education or continuing education, 46% employed, 59% cohabiting, 85% with alcohol dependence diagnosis | Patients attending public outpatient’s alcohol clinic in Odense; aged≥18 years, harmful alcohol use or alcohol dependence syndrome according to the International Classification of Diseases, Tenth Revision (ICD-10), and written informed consent; 60% participation rate; 63% at 3 months, 38% at 6 months, 66% at 12 months | Video conferencing; usual face-to-face care with the option of opt in videoconferencing. Received between 1 and 3 sessions a week at the initial stages followed by 1 session every other week for about 7 months with optional video conferencing (TAU+I); 50% of the intervention group used videoconferencing for a total of 60 treatment sessions. Duration of video sessions not stated. 62% of sessions had technical problems | Face-to-face; TAU, ie, usual face-to-face care for up to 7 months; received between 1 and 3 sessions a week at the initial stages of treatment, followed by 1 session every other week for about 7 months; not stated | Not stated | Change in days of alcohol use in the past month from baseline to 12 months follow-up: TAU+I, mean=-13.75 (SD 12.40), TAU, mean=-12.44 (SD 10.53) (NS). Change in days of excessive alcohol use in the past month from baseline to 12 months follow-up: TAU+I, mean=-13.47 (SD 11.79), TAU, mean=-12.26 (SD 11.29) (NS); not stated |

^a^NS: no significant difference.

^b^AUDIT: Alcohol Use Disorders Identification Test.

^c^RAPI: Rutgers Alcohol Problem Index.

^d^MET: motivational enhancement therapy.

^e^AIRR: adjusted incidence rate ratio.

^f^TAU: treatment as usual.
