# Supplementary material for: Effectiveness of Individual Real-Time Video Counseling on Smoking, Nutrition, Alcohol, Physical Activity, and Obesity Health Risks: Systematic Review
Source: J Med Internet Res. 2020 Sep 11;22(9):e18621. doi: 10.2196/18621 (PMC7519427; doi:10.2196/18621)
Supplement: Multimedia Appendix 3 [file jmir_v22i9e18621_app3.docx]

**Table 3.** Characteristics of studies examining the effectiveness of video counseling on physical activity.

| Author; country; years data collected; source of funding | Study design; setting | Population; sample characteristics | Recruitment method; eligibility criteria; participation rate; retention at follow-up | Video intervention mode; video intervention description; video intervention received | Comparator mode; comparator description; comparator received | Satisfaction measures | Outcome measures; cost |
| --- | --- | --- | --- | --- | --- | --- | --- |
| Alley et al [77]; Australia; 2014–2015; National Health and Medical Research Council of Australia and the National Heart Foundation of Australia | 3-arm randomized trial of tailoring+video coaching, tailoring-only, or waitlist control group; participants recruited from metropolitan and regional areas | Nonclinical; N=154, 76% female, mean age 54 years, 43% not paid employment, 82.8% completed higher education, 39.7% physically active, average time for physical activity 168 min/week, average BMI 31 kg/m^2^ (obese range), average mental health score 43.4 | Print (newspaper ads, posters, and leaflets articles) and web advertising (Google and Facebook); Australian, >18 years old, English speaking; 63% participation rate; 55% at 9 weeks, 38% at 6 months | Skype video coaching; tailoring+video coaching, 8-week computer-tailored web-based physical activity intervention (“My Activity Coach”) and 4 10-min coaching sessions using the video-calling program Skype every 2 weeks; 21% completed 1 video session. Of those participants, an average of 2.4 video sessions were completed. Average video session length was 10.4 min | Web based, none (waitlist); a computer-tailored web-based physical activity intervention, emails (tailored advice, with graphs and minimum and optimal physical activity recommendations), a waitlist control group; mean time spent on website=75.25 min (SD 52.90), not applicable | NS^a^ between tailoring+video coaching vs tailoring-only groups on program satisfaction scores (NS) | Change in physical activity (min/week) from baseline to week 9: tailoring+video coaching vs control, estimate 140.94 (-254.01 to -27.87) (S^b^). tailoring+video coaching vs tailoring-only, estimate 35.39 (-148.50 to 77.71; NS); change in physical activity (min/week) from baseline to 6 months: tailoring+video coaching vs control, estimate 66.16 (-244.55 to 112.24) (NS), tailoring+video coaching vs tailoring-only, estimate -25.16 (-211.74 to 161.43) (NS); not stated |
| Chemtob et al [79]; Canada; 2016-2017; Craig H. Neilsen Foundation | 2-arm randomized trial with video counseling vs no counseling (regular daily routine); adult patients with SCI^c^ from rehabilitation hospitals in Montreal | Clinical; n=24; 95.5% white, 86.4% Canadian, 72.7% male, mean age 51.6 years, 72.7% married or common law, 31.8% employed, mean years since SCI 15.45 | Participants were recruited from outpatient rehabilitation hospitals in Montreal, a local adapted fitness center, an organization representing persons with SCI, pre-existing databases of previous research participants, and social media platforms; aged ≥18 years, have paraplegia and use a mobility device, sustained a SCI at least 1 year prior, be minimally active (engaging in <2 bouts of LTPA^d^ per week in the last 2 months), speak and understand English or French, had the intention to become physically active in the next 2 months, have access to a computer that meets the specific software requirements used in the intervention, and access to internet with adequate bandwidth (or a stable 3G cellular connection for wireless internet); 40% participation rate; 92% retention rate at 10 weeks | Video counseling using remote education, augmented communication, training and supervision; starting 2 weeks from baseline, the LTPA counselor conducted 1 LTPA counseling session per week for 8 weeks, resulting in a total of 8 counseling sessions; video sessions received not stated | Regular daily routine (no counseling); regular daily routine with no encouragement to increase or reduce current physical activity levels; not stated | Not stated | LTPA: compared with the control group, the video counseling group reported greater total minutes of LTPA at 6 weeks (Hedge g=0.87) and 10 weeks (Hedges=0.85). From baseline to 6 weeks: 70% in the video counseling group compared with 50% in the control group increased their total levels of LTPA by >20 min. From baseline to 10 weeks: 90% of participants in the video counseling group and 50% of participants in the control group increased their total levels of LTPA by >20 min; MVPA^e^: moderate effect sizes were found at 6 weeks (Hedge g=0.52) and small effect sizes were found at 10 weeks (Hedge g=0.34) favoring the video counseling group (M6 weeks=168.72, SD6 weeks=221.22) over the control group (M6 weeks=81.54, SD 6 weeks=118.68). From baseline to 6 weeks: 70% in the video counseling group compared with 50% in the control group increased their MVPA by >20 min. From baseline to 10 weeks: 70% of participants in the video counseling group and 42% of participants in the control group increased their total levels of MVPA by >20 min. |
| Weinstock et al [78]; USA; 2000–2007; Centers for Medicare and Medicaid Services | 2-arm randomized trial of usual care vs a telemedicine intervention; PCP^f^ practices, half urban and half rural | Clinical; N=1650, age range 65-80 years, 63% female, 35% Hispanic, mean BMI 32 kg/m^2^. Telemedicine group: 41% married/living with significant other, mean years of education 38, mean days of physical activity 2.8. Usual care group: 41.1% married/living with significant other, mean years of education 36.8, mean days of physical activity 2.9 | Participants were recruited through their PCP practice; Medicare beneficiaries aged ≥55 years, fluent in either English or Spanish, have diabetes mellitus and live in a federally designated medically underserved area or health professional shortage areas. The urban component enrolled patients living in Harlem, Washington Heights, and Inwood in northern Manhattan; participation rate not clear; not stated | Video counseling via a home telemedicine unit; home video visits with diabetes educator every 4-6 weeks for self-management report and daily pedometer use for 5 years; not stated | Face-to-face care; participants received usual clinic-based care delivered face-to-face; not stated | Not stated | Physical activity across 5-year period: lower rate of decline in physical activity over time in the video counseling group (0.49) than in the usual care group (0.83) for a difference of 0.34 (CI=0.02, 0.66). (S); not stated |

^a^NS: no significant difference.

^b^S: significant difference.

^c^SCI: spinal cord injury.

^d^LTPA: leisure time physical activity.

^e^MVPA: moderate and vigorous physical activity.

^f^PCP: primary care provider.
