# Supplementary material for: Effectiveness of Individual Real-Time Video Counseling on Smoking, Nutrition, Alcohol, Physical Activity, and Obesity Health Risks: Systematic Review
Source: J Med Internet Res. 2020 Sep 11;22(9):e18621. doi: 10.2196/18621 (PMC7519427; doi:10.2196/18621)
Supplement: Multimedia Appendix 4 [file jmir_v22i9e18621_app4.docx]

**Table 4.** Characteristics of studies examining the effectiveness of video counseling on obesity.

| Author; country; years data collected; source of funding | Study design; setting | Population; sample characteristics | Recruitment method; eligibility criteria; participation rate; retention at follow-up | Video intervention mode; video intervention description; video intervention received | Comparator mode; comparator description; comparator received | Satisfaction measures | Outcome measures; cost |
| --- | --- | --- | --- | --- | --- | --- | --- |
| Hansen et al [80]; Denmark; 2012–2015; Capital Region of Denmark, the City of Copenhagen, Smedemester Niels Hansens og hustru Frederikkes Fund | 2-arm randomized trial of a video consultation intervention vs standard care; outpatient department in 3 hospitals | Clinical; N=165, mean age 58 years, 64% males, 28% nonwestern background, 23% unemployed | An endocrinologist and a study nurse recruited eligible participants from an outpatient department via telephone, written, and face-to-face; Danish-speaking inhabitants of the City of Copenhagen aged 30-75 years, with history of type 2 diabetes. HbA_1c_ >7.5% (59 mmol/mol), BMI >25 kg/m^2^; 19% participation rate; 88% retention at 8 months, 84% retention at 14 months | Video conference via a tablet computer; video add-on: videoconferences in usual clinic-based care with health care center nurse via tablet for 32 weeks. Participants upload blood sugar at 16, 32, and 58 weeks and blood pressure and weight (baseline and after 32 weeks) via Bluetooth or USB; not stated | Face-to-face care only (ie, usual care); usual clinic-based visits at the General Practitioner or diabetes clinic every 3-6 months; not stated | Not stated | No changes in BMI or waist/hip ratio; not stated |
| Homma et al [81]; Japan; 2012; Ministry of Education, Culture, Sports, Science and Technology | 2-arm cluster randomized trial of individualized monthly documented reports vs interactive videophone communication; urihara city (2 city districts) | Clinical: n=68. Document group: n=33, mean age 67 years, female 67%, mean BMI (kg/m^2^) 25.0, average steps per day 5046. Videophone group: n=35, mean age 65 years, female 63%, mean BMI (kg/m^2^) 24.3, average steps per day 5829 | Advertisement in community paper and community leaflet distributed by community leader; individuals with lifestyle disease, eg, hypertension, diabetes, obesity, and those susceptible to these such as insufficient exercise levels; not clear; 97% retention at 3 months | Interactive video phone via 2-way interactive video phone (VP-2000, NTT East Japan)+pedometer; video consultations were delivered 3 times in 3 months (ie, at 2 weeks, 1.5 months, and 1 week to final monitoring). 20 min for the first and last sessions and 15 min for the intervening session; not stated | Individualized documented reports at 3 time points; documented reports involved graphical representation of telemonitoring data, blood pressure, body weight, monthly average steps per day, instructions for lifestyle modification, and blood tests as well as anthropometric measurements. Delivered at 2 weeks, 1.5 months, and 1 week to final monitoring; not stated | Not stated | Change in BMI from preintervention to postintervention (3 months): video, pre - 24.3 (SE=0.5), post - 24.1 (SE=0.5), reports, pre - 25.0 (SE=0.6), post - 24.4 (SE=0.6), significant difference between groups. Change in average steps/day from preintervention to postintervention (3 months): video, pre - 5829 (SE=423), post - 7324 (SE=483), reports, pre - 5046 (SE=454), post - 5992 (SE=424), no significant difference between groups; not stated |
| Johnson et al [82] USA; year data collected not stated; National Institute of General Medical Sciences | 3-arm randomized trial of videoconferencing vs the in-person and control groups; patients with BMI ≥30 kg/m^2^. No other setting reported | Clinical; n=30. Video conference group: n=10, mean age: 43 years, mean weight: 112.8 kg, mean BMI: 38.6 kg/m^2^ mean steps/day: 3755.1. In-person group: n=10, mean age: 42.2 years, mean weight: 101.5 kg, mean BMI: 35.3 kg/m^2^, mean steps/day: 3641.7. Control group: n=10, mean age: 44.5 years, mean weight: 95.9 kg, mean BMI: 34.5 kg/m^2^, mean steps/day: 4324.3 | Not clear; participants had BMI ≥30 kg/m^2^, English speaking, nondiabetic, ambulatory, <396 pounds in weight, a sedentary lifestyle (<7000 steps/day), not regularly engaging in moderate intensity activities, owners of an iPhone or Android smartphone and able to travel for scheduled appointments; not clear; 100% | Video conferencing; m-health device data uploads (via Withing app, Healow app) received educational materials, weekly, individualized video conferencing health coaching by a multidisciplinary team (registered dietitian, exercise physiologist, and medical doctor) based on data uploaded over the 12-week intervention. During the dietitian sessions, daily and weekly caloric intakes retrieved from the MyFitnessPal app were reviewed, discussed, and adjustments made as needed. During the sessions with the exercise physiologist, discussions included current exercise routine, goal setting, and physical activity progression (ie, more steps/day, more minutes/day). The project’s medical doctor oversaw all dietary and exercise recommendations; not stated | In-person, control (m-health); in-person received individualized health coaching by a multidisciplinary team (registered dietitian, exercise physiologist, and medical doctor) based on data uploaded over the 12-week intervention. Control group received m-health devices, no health coaching sessions or feedback on steps per day from the Withings watch or calories entered into MyFitnessPal; not clear | Not stated | Body weight loss: the videoconferencing group achieved a significantly greater weight loss (8.23 kg SD 4.5) from baseline to 12 weeks than the in-person group (3.2 kg SD 4.5) and control group (2.9 kg SD 3.9) (significant difference). Steps per day: videoconferencing had significantly higher steps/day than in-person at week 4 by 1063 steps/day and control group at week 6 by 2107 steps/day, week 8 by 1883 steps/day, week 9 by 2318 steps/day, and week 11 by 1961 steps/day (significant difference); not stated |
